# MR Imaging in Diagnosis of Pelvic Floor Descent: Supine versus Sitting Position

**DOI:** 10.1155/2016/6594152

**Published:** 2016-01-12

**Authors:** Francesca Iacobellis, Antonio Brillantino, Adolfo Renzi, Luigi Monaco, Nicola Serra, Beatrice Feragalli, Aniello Iacomino, Luca Brunese, Salvatore Cappabianca

**Affiliations:** ^1^Department of Radiology, Second University of Naples, Piazza Miraglia 2, 80138 Napoli, Italy; ^2^“Villa delle Querce” Hospital, Via Battistello Caracciolo 48, 80136 Napoli, Italy; ^3^“Villa Esther” Hospital, Via Due Principati 169, 83100 Avellino, Italy; ^4^Department of Medical, Oral and Biotechnological Sciences, “G. D'Annunzio” University, Via dei Vestini, 66013 Chieti, Italy; ^5^Department of Health Science, University of Molise, Viale Giovanni Paolo II 1, 86100 Campobasso, Italy

## Abstract

*Introduction*. Functional disorders of the pelvic floor represent have a significant impact on the quality of life. The advent of open-configuration systems allowed for the evaluation of defecation with MR imaging in sitting position. The purpose of the present study is to compare the results of static and dynamic pelvic MR performed in supine position versus sitting position, using a new MR prototype machine, in the diagnosis of pelvic floor descent. *Materials and Methods*. Thirty-one patients with pelvic floor disorders were enrolled, and underwent MR Defecography in supine position with 1.5 T closed magnet (MAGNETOM Symphony, Siemens, Germany) and in sitting position with a 0.25-Tesla open magnet system (G-Scan ESAOTE, Italy). *Results*. In rest and squeezing phases, positions of bladder, vagina, and ARJ were significantly different when the patient was imaged in supine versus sitting position. In the defecation phase, a significant difference for the bladder and vagina position was detected between the two exams whereas a significant difference for the ARJ was not found. A statistically significant difference exists when the pelvic floor descent is evaluated in sitting versus supine position. *Conclusion*. Our results show that MR Defecography in sitting position may represent a useful tool to correctly diagnose and grade the pelvic organ descent.

## 1. Introduction

Functional disorders of the pelvic floor represent common clinical problems and have a significant impact on the quality of life. They comprise a wide range of clinical conditions, including urinary incontinence, sensory and emptying abnormalities of the lower urinary tract, fecal incontinence, defecatory dysfunction, chronic pelvic pain syndromes, and pelvic organ prolapse [1, 2]. Pelvic floor disorders often coexist and, therefore, incontinence, descensus, and organ prolapse may occur in many different combinations [3–5]. Risk factors for pelvic floor dysfunction include pregnancy, multiparity, advanced age, menopause, obesity, connective tissue disorders, smoking, and chronic obstructive pulmonary disease, as well as any other component that results in a chronic rise in intra-abdominal pressure [2, 6, 7]. Although the collection of the clinical history and the physical examination represent the first step in the evaluation of patients with pelvic floor dysfunctions [8], a multidisciplinary approach and the employment of panoramic radiological investigations with a wide and detailed view of the pelvis are needed for a more detailed diagnosis and grading of pelvic floor disorders [2, 9–11] and for the surgical planning [12–16].

Weakness of the pelvic floor can involve anterior, middle, and posterior compartments, producing an abnormal descent of the bladder, uterus, and bowel.

In case of pelvic floor weakness, traditionally diagnosed via physical exam, pelvic magnetic resonance (MR) imaging, with its superior soft-tissue contrast resolution, allows direct visualisation of the pelvic organs and their supportive structures in a single, dynamic, and noninvasive examination [1, 3, 6, 7, 10, 17]; the supine position of the patient during the examination may be a disadvantage, because it may influence the pelvic floor physiology as well as the dynamic defecation process [1, 3].

The advent of open-configuration systems allowed the evaluation of defecation with MR imaging in sitting position, and several studies were performed [5, 18–23]. However, the magnet configuration and the examination technique, as well as the accuracy of the sitting position in the diagnosis of the pelvic floor disorders, are not standardised, not completely defined, and currently debated in literature.

The purpose of the present study is to compare the results of static and dynamic pelvic MR performed in supine position versus sitting position, using a new MR prototype machine, in the diagnosis of pelvic floor descent.

## 2. Materials and Methods

### 2.1. Patients and Methods

#### 2.1.1. Ethics

The study was approved by the institutional ethical committee. All patients gave their written informed consent to take part in this study.

From January 2012 to December 2014, all the patients referring to our Radiology Department for pelvic dynamic MRI for the evaluation of pelvic floor disorders were investigated about their clinical history and considered for enrolment in this study.

All the patients eligible for their physical prerequisites (hip circumference less than 100 cm) were asked to be enrolled in the study and so patients that gave their consent underwent sitting MR examination after supine MR examination.

#### 2.1.2. MRI Technique

MR images were obtained after administration of contrast agent (ultrasound gel) into the rectum and vagina in both sitting and supine positions. To ensure an adequate bladder filling, all patients were invited to drink 500–700 mL of water 15–20 min before the examination. Rectum and vagina were filled with 200 mL and about 25–30 mL, respectively, of ultrasonographic gel (Aquasonic, Parker Laboratories, Fairfield, NJ, USA). Rectal cleaning was considered unnecessary.

*1.5 T Dynamic MR Defecography*. All supine imaging studies were performed on 1.5 T closed magnet (MAGNETOM Symphony, Siemens, Germany). All the patients were supine imaged with a four-channel body-phased-array receiver coil.

After an initial localizer in three different planes, the study protocol includes the following morphological (static) sequences: axial TSE T1-W (TR/TE 611/11; slices: 25; thickness: 5 mm; matrix: 256 × 256; flip angle: 150°), axial TSE T2-W (TR/TE 6430/114; slices: 25; thickness: 5 mm; matrix: 256 × 256; flip angle: 180°), and sagittal TSE T2-W (TR/TE 4650/127; matrix: 256 × 256; slices: 20; thickness: 4 mm; flip angle: 150°).

Functional dynamic sequences TRUE FISP T2-W sagittal (TR/TE 3.75/1.6; matrix: 256 × 256; slices: 1; thickness: 8 mm; flip angle: 80°) during maximal pelvic floor contraction (squeezing) and defecation phases were acquired. During the dynamic sequences of the examination, patients were instructed via headphones: they were asked first to squeeze and after to strain emptying the rectum as completely as possible. The MR-D images so obtained were assembled in cineview in postprocessing. Examination time (static and dynamic sequences) took about 25–30 min to be completed.

*0.25 T Open Magnet MR Defecography*. After the examination in supine position, patients were transferred to a 0.25-Tesla open magnet system (G-Scan ESAOTE) and underwent the examination in sitting position.

**Figure 1:**
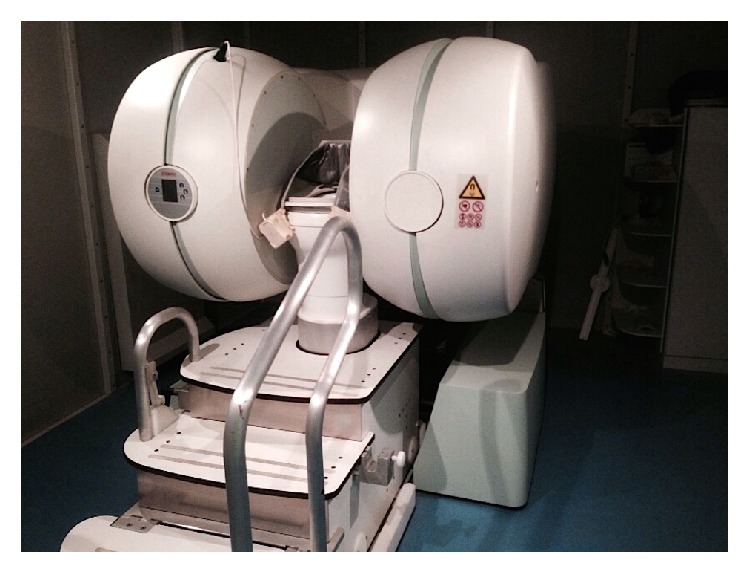
“Pelvic scan” prototype.

The adopted magnet is a prototype made modifying the G-Scan ESAOTE tilting open magnet system to carry out the examination with the patient in sitting position on a dedicated commode (Figure 1).

The G-Scan ESAOTE MRI system was originally designed to study the joints and the spine, either in a clinostatic (supine) or in an orthostatic (weight-bearing) position since magnet and patient can rotate from 0 to 90 degrees.

The prototype available in our institution was obtained, positioning the magnet at 90 degrees, increasing the distance originally existing in the G-Scan ESAOTE magnet to insert a dedicated commode equipped with a flexible single channel receiving coil. The coils were specifically designed to maximize the signal/noise ratio in the pelvic floor and they consist of a belt part with solenoidal coils arranged to optimize the signal reception from the lower trunk area, connected to a surface part with concentric coils allowing us to detect signal from the lower part of the pelvic floor. The coils were realized in two different lengths: small, 96 cm, and large, 116 cm.

This allowed patients to be studied in the physiological position adopted during defecation.

The sequence adopted for the dynamic study (2D HYCE sagittal) was specifically developed for this new prototype and it is a balanced steady-state gradient-echo sequence that allows one to acquire images of the same layer previously selected by the user, repeatedly.

The rectum and vagina were filled of gel and static images were first obtained acquiring the following sequences: axial FSE T2 (TR/TE/NEX, 3140/100/1; slices: 19; thickness: 6 mm; FOV: 420*∗*420; oversampling: 130), sagittal FSE T2 (TR/TE/NEX, 3200/100/1; slices: 11; thickness: 6 mm; FOV: 300*∗*300; oversampling: 182). During squeezing and defecation, functional 2D HYCE sagittal (TR/TE/NEX 14/7/1; slices: 1, thickness: 12.5 mm, FOV: 280*∗*280, matrix: 208*∗*206) sequences were acquired in sitting position. Overall MR time for the study was approximately 25–30 minutes.

#### 2.1.3. Image Analysis

Images were analysed in consensus by an experienced board-certified abdominal radiologist (SC) and a radiology resident with four years of experience in abdominal radiology (FI).

The degree of the pelvic organs descent was evaluated measuring the perpendicular distance between the pubococcygeal plane (PCP) and the bladder base and the posterior vaginal fornix or the vaginal vault (if the patient was hysterectomized) and the anorectal junction (ARJ) during each of the three phases: rest, squeezing, and defecation in both supine and sitting MR examinations. The reference plane used for MRI, the PCP, is defined as the plane of the pubococcygeal line (PCL) which connects the inferior margin of the symphysis pubis with the last coccygeal joint. The anorectal junction is defined as the point of taper of the distal part of the rectum as it meets the anal canal, corresponding to the posterior impression of the transition between puborectal muscle and levator plate, and it represents the point of reference for posterior compartment descent [24, 25].

According to the majority of the authors, an ARJ position lower than 3 centimetres (cm) in respect to PCP in the resting phase or a descent of more than 3 cm during the evacuation, if compared with the position at rest, is the definition of fixed and dynamic perineal descent, respectively [5, 26–30].

A descent of more than 1 cm at rest or during evacuation of the bladder base and of the posterior vaginal fornix or vaginal vault in respect to the PCP is considered suggestive for anterior and middle prolapse, respectively [5, 25]. The distances in centimetres between PCP and bladder and vaginal fornix or vaginal vault and ARJ were considered positive if they have a position above PCP, negative if they have a position under PCP, and null value if they have a position on the PCP.

Data were compared analysing the difference between the two different positions in the three different phases (rest, squeeze, and defecation), the difference in the detection of fixed and dynamic perineal descent, and the existence of possible correlation between supine and sitting positions.

#### 2.1.4. Statistical Analysis

The statistical analyses were performed using MATLAB statistical toolbox version 2008 (MathWorks, Natick, MA, USA) for Windows at 32 bits on random sample of 31 patients, 12.90% males and 87.10% females. ANOVA test [31], Fisher's exact test, Pearson linear correlation [32], Student *t*-test, and *Z*-test [33] were used for data analysis. A *p* value < 0.05 was considered significant.

## 3. Results

Two hundred patients with clinical symptoms suggestive for pelvic floor descent referred to our Radiology Department for pelvic dynamic MRI for the evaluation of pelvic floor disorders.

Fifty patients satisfied the physical prerequisite to be examined in sitting position and they were asked to take part in the study.

Out of these, 31 patients (27 female, 473 male; mean age: 48.5 years; range: 21–74) gave the consent to participate in the study and were imaged in both positions.

The procedures were well tolerated by all the patients and were successful in all cases. The average total examinations time was 60 minutes per patient.

In all cases, the images quality was diagnostic.

In Table 1, the measures (in centimetres) of pelvic organs in respect to the PCP, in rest, squeeze, and defecation phases, in both sitting and supine examinations in all patients are reported.

**Figure 2:**
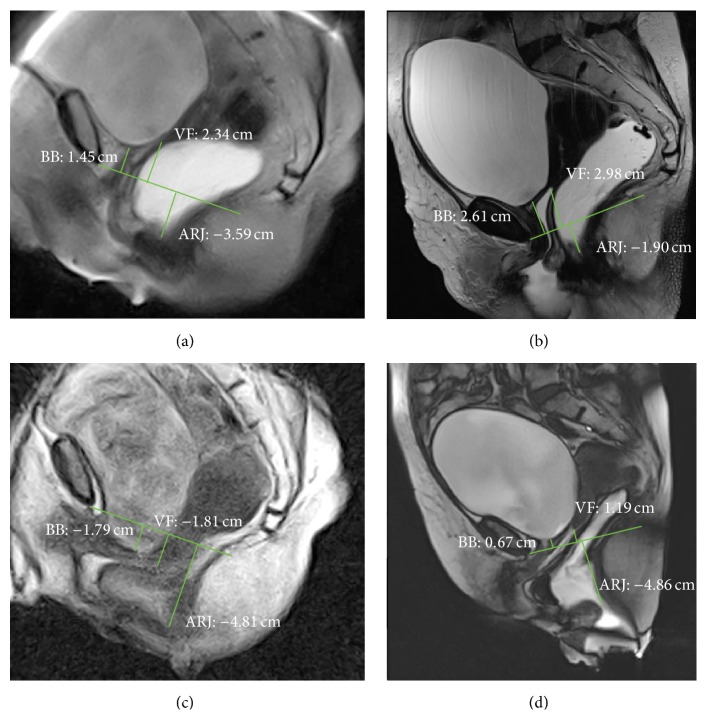
MR Defecography. Rest phase in sitting (a) and supine (b) position. Evacuation phase in sitting (c) and supine (d) position. The pathological fixed descent was detected only in sitting position in rest phase (a). In evacuation phase, a cystocele became evident (d), whereas the maximal descent of the ARJ is similar in both sitting and supine position (c, d). BB: bladder base; VF: vaginal fornix; ARJ: anorectal junction.

**Figure 3:**
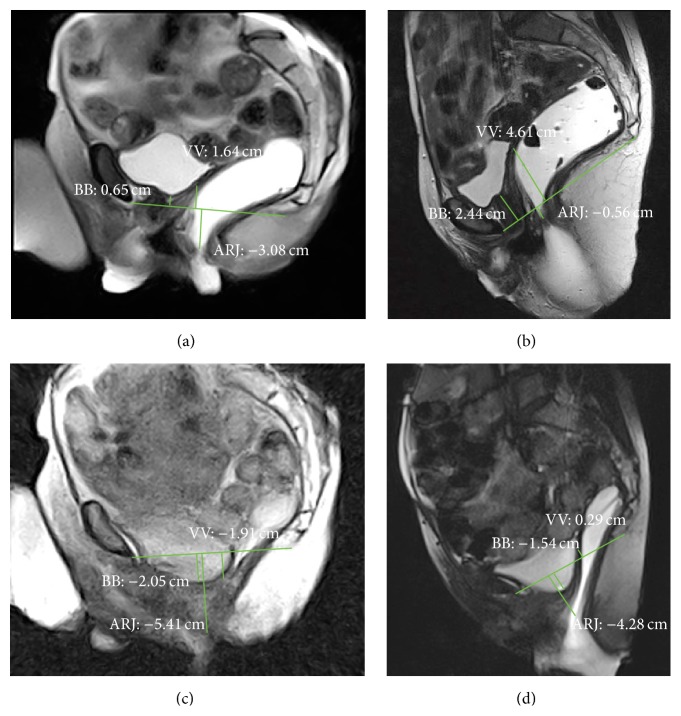
MR Defecography. Rest phase in sitting (a) and supine (b) position. Evacuation phase in sitting (c) and supine (d) position. The pathological fixed descent was detected only in sitting position in rest phase (a). In evacuation phase, a cystocele and a vaginal vault prolapse became evident (c), and the MR examination in supine position overestimates the dynamic descent, nonpathological in (a) and (c) and pathological in (b) and (d). BB: bladder base; VV: vaginal vault; ARJ: anorectal junction.

**Figure 4:**
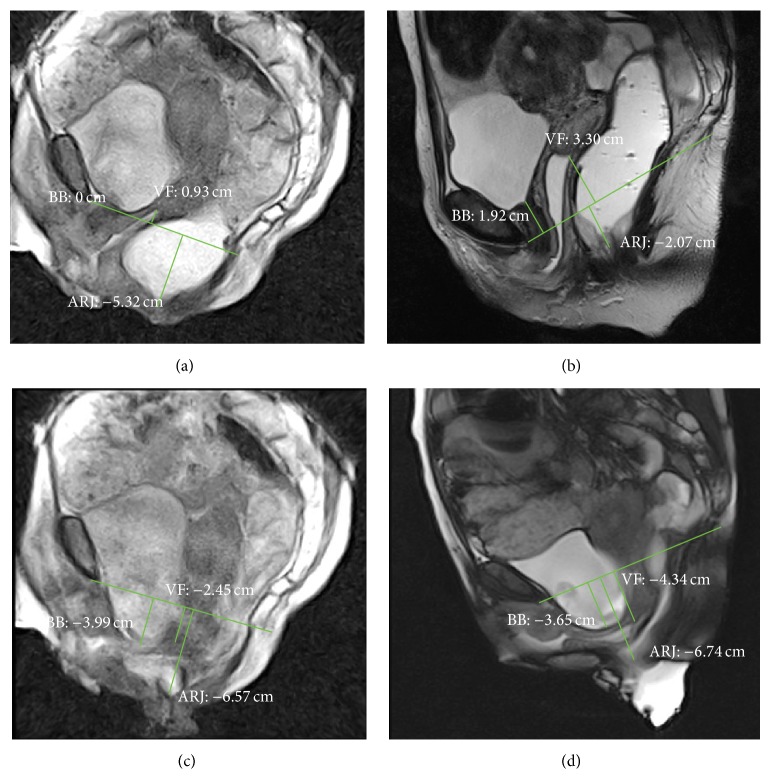
MR Defecography. Rest phase in sitting (a) and supine (b) position. Evacuation phase in sitting (c) and supine (d) position. The pathological fixed descent was detected only in sitting position in rest phase (a). In evacuation phase, the MR examination in supine position overestimates the dynamic descent; the rectocele is seen only in sitting position. BB: bladder base; VF: vaginal fornix; ARJ: anorectal junction.

In rest phase, both positions of bladder and ARJ were significantly different when the patient was imaged in supine versus sitting position (*p* value ≤ 0.0001 and *p* value ≤ 0.001, resp.) (Figures 2(a), 2(b), 3(a), and 3(b)); also during squeezing, both positions of bladder and ARJ were significantly different when the patient was imaged in supine versus sitting position (*p* value = 0.0011; *p* value = 0.0154). In the defecation phase, a significant difference for the bladder position was detected between the two exams (*p* value ≤ 0.001) whereas a significant difference for the ARJ was not found (*p* value = 0.373) (Figures 2(c), 2(d), 4(c), and 4(d)).

In the rest phase, a fixed pelvic floor descent was detected in sitting position in 16/31 (51.6%) patients whereas only in 2/31 (0.64%) the supine MR detected a descent of more than 3 cm (*p* > 0.0005) (Figures 2(a), 2(b), 3(a), 3(b), 4(a), and 4(b)).

In rest phase, a cystocele was detected in sitting position in 4/31 (12.9%) patients whereas in 0/31 (0%) the supine MR detected a descent of more than 1 cm (*p* > 0.11).

In evacuation phase, a cystocele was detected in sitting position in 20/31 (64.5%) patients whereas in 14/31 (45.16%) the supine MR detected a descent of more than 1 cm (*p* = 0.20) (Figures 3(c) and 3(d)). The dynamic descent for the bladder and the ARJ was also evaluated and compared: a statistically major descent was detected in supine position if compared with sitting position for both bladder and the ARJ (*p* value = 0.04; *p* value = 0.0157) (Figure 2).

**Figure 5:**
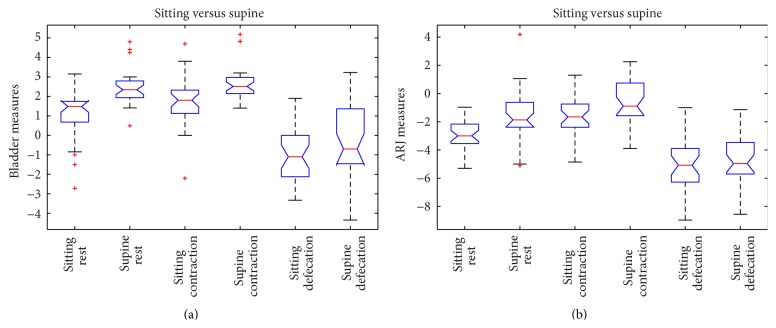
ANOVA box plot for bladder (a) and ARJ (b) measures of all the patients.

In Figure 5, the graphic representation of the ANOVA test is shown.

A dynamic descent was detected in sitting position in 10/31 (32.25%) patients and in 18/31 (58%) in supine position (*p* = 0.3).

The measures of pelvic organs in respect to the PCP were also examined for the female and male subgroups as shown in Table 2.

In the female subgroup (*n* = 27), in rest phase, the positions of bladder, ARJ, and vagina were significantly different (*p* value ≤ 0.0001) when the patient is imaged in supine versus sitting position.

In squeezing phase only for bladder and vagina, there was a statistically significant difference (*p* value = 0.0002 and *p* value = 0.0013) whereas for ARJ measure a significant difference was not detected with a probability more than or equal to 95% (*p* value = 0.0735). In the defecation phase for ARJ measures, a significant difference was not detected (*p* value = 0.572), whereas there was a statistically significant difference for bladder and vagina measures (*p* values < 0.001; *p* value = 0.019).

The dynamic descent of bladder, ARJ, and vagina between rest and defecation phases in both positions was also compared and for the ARJ a statistically significant major descent was detected in supine position versus sitting position (*p* value = 0.018). A significant difference between sitting and supine positions in the degree of descent of the bladder (*p* value = 0.0239) was found; a significant difference was not detected for the vagina measure with a probability more than or equal to 95% (*p* value = 0.278).

**Figure 6:**
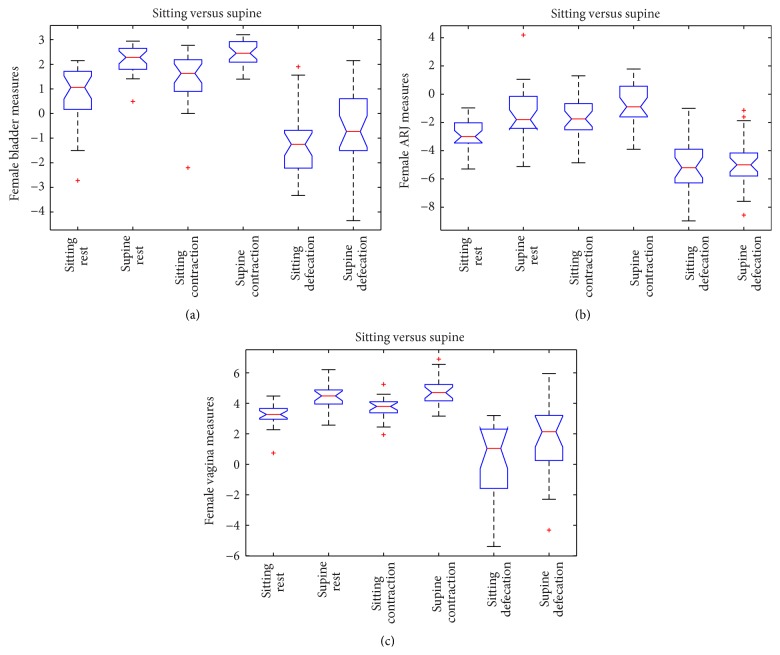
ANOVA box plot for bladder (a), vagina (b), and ARJ (c) measures of the female subgroup.

In Figure 6, the graphic representation of the ANOVA test for the female subgroup is shown.

In the male subgroup (reported for completeness, *n* = 4), in rest phase, there was a statistically significant difference in the bladder measures between supine and sitting positions (*p* value = 0.0217), whereas there were not significant differences for ARJ measures (*p* value = 0.09) between supine and sitting positions.

In squeezing phase, significant differences for bladder and ARJ measures between supine and sitting positions were not found (*p* value = 0.346 and *p* value = 0.124, resp.).

In the defecation phase, significant differences for both bladder and ARJ measures between supine and sitting positions were not found (*p* value = 0.066 and *p* value = 0.297, resp.).

The dynamic descent of the bladder and the ARJ between rest and defecation phases in both positions was also compared and statistically significant differences were not found for both bladder and ARJ (*p* value = 0.906 and *p* value = 0.982, resp.).

The results of the Pearson correlation test are shown in Table 3.

**Figure 7:**
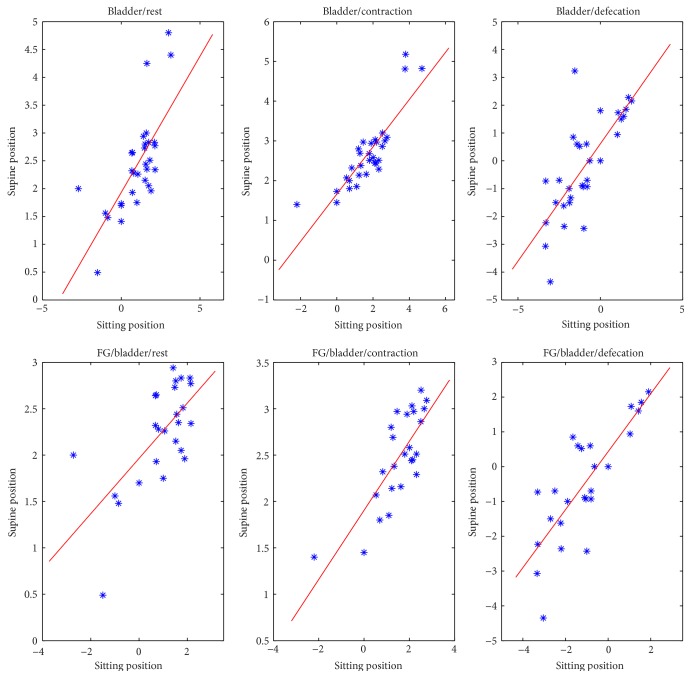
Strong linear correlation for bladder measures in all phases between sitting and supine positions, for all patients.

A strong linear correlation in the bladder measures detected in sitting and supine MR examination was found in all phases (rest, squeeze, and defecation) (Figure 7).

**Figure 8:**
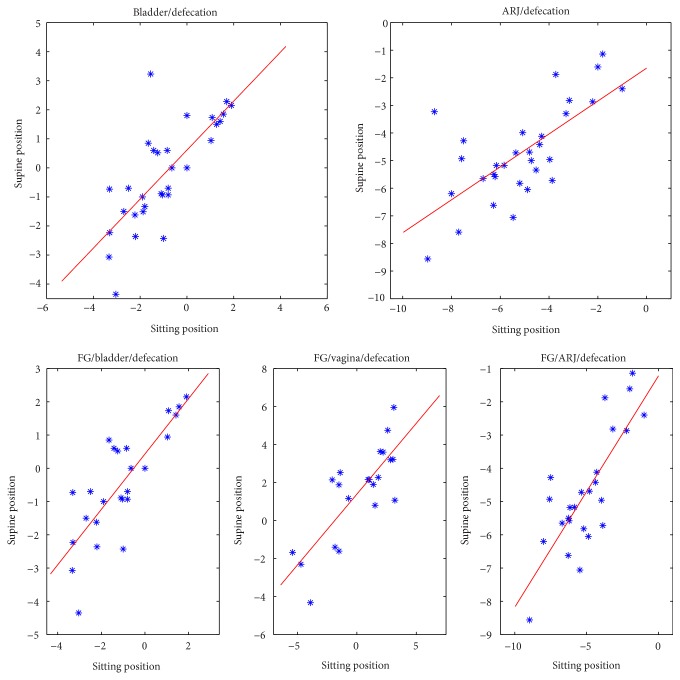
Linear correlation graphs of bladder and ARJ measures on all patients and of bladder, vagina, and ARJ measures on FG in defecation phase between sitting and supine positions.

In the female subgroup, a moderate correlation was found for the vagina measures in rest phase and a strong correlation was detected in defecation phase (Figure 8).

A strong correlation for the ARJ measure was found in defecation phase, whereas it was weak in rest phase (Figure 8).

## 4. Discussion

Weakening of the pelvic floor is a debilitating disorder usually involving middle-aged and elderly parous women, even if pelvic floor disorders may also occur in male patients [23, 34, 35]. Weakening of the pelvic floor may result in an abnormal descent of the bladder, the uterus, or the vaginal vault and the rectum, with pelvic organ prolapse and related symptoms including urinary incontinence, fecal incontinence, or obstructed defecation syndrome. The diagnostic limitation of the pelvic examination alone has led to the need of using more direct and comprehensive diagnostic methods [3]. In the assessment of patient with pelvic floor disease, several radiological investigations are used [9]: RX-Defecography is considered the “gold standard” in the evaluation of pelvic floor diseases, being a cost-effective procedure, easy to perform, and widely available. However, it is an invasive procedure due to the ionizing radiations and the administration of four contrasts and it allows one to evaluate only the opacified organs, neither muscular structures nor soft tissues of the pelvic floor [36]. Ultrasound (US) has the advantage of the lack of ionizing radiation, but this method has several limitations in evaluating pelvic organs prolapse [2]. The alternative, especially in complex combined pelvic floor disorders, is represented by dynamic MR, first described by Yang et al. in 1991, that allows for a multiplanar and multiparametric evaluation of the three pelvic compartments (anterior urinary, middle genital, and posterior digestive) and the direct and detailed visualization of the pelvic floor structures without using ionizing radiation because of its intrinsic soft-tissue contrast capability [3, 4, 12, 37, 38].

In the axial, T1 and T2 weighted, and sagittal, T2 weighted, dynamic sequences, the three different pelvic compartments are displayed to evaluate their morphology and signal characteristics and their position across the different phases (rest, straining, and evacuation) in respect to the PCL with a real-time evaluation of patterns of dysfunction; the supporting ligaments and the muscles can be adequately investigated to detect if there are associated muscular and fascial defects, thus providing the surgeon with a road map for tailored treatment. The assessment of the peritoneal compartment (the fourth pelvic compartment) is important especially for the surgical planning and it appears clearly visible on MRI as a thin, low signal band, outlined by the high signal of fat [37].

MR evaluation of pelvic floor descent is limited by the closed architecture of conventional MR systems allowing the patient to be examined only in supine position. Pelvic floor abnormalities may not be detected or misinterpreted if the examination is not completed with evacuation phase; this can be difficult to perform in supine position, limiting the diagnosis [3, 39].

The availability of open magnet systems allows us to perform MR Defecography in sitting position: this is an ideal tool to assess pelvic floor disorders in a physiological position with the advantage of good delineation of all pelvic soft tissues [3]. The use of this technique is limited by worldwide availability. Some authors reported that to perform the examination using a state-of-the-art technique, which means dynamic MR imaging in supine position in closed magnet at rest, during squeezing, straining, and evacuation is probably more important than to consider the patient position [11, 17, 40, 41]. In our previous experience, imaging the patients in supine position has been shown to be satisfactory in the evaluation of symptomatic pelvic floor weakness even if defects are best demonstrated when patients are sitting [42, 43]. According to this, the results of the present study show that a statistically significant difference exists when the pelvic floor descent is evaluated in sitting versus supine position, and the MR study in supine position can underestimate the fixed descent (Figures 2, 3, and 4). In our series, the percentage of patients with a pathological fixed pelvic floor descent (ARJ more than 3 cm below the PCP) evaluated in rest phase significantly differs between the two procedures.

No significant differences were found in the percentage of patients with cystocele detected in sitting position versus supine position at rest, even if the positions of the bladder significantly differ when the patient is imaged in supine versus sitting position.

In defecation phase no significant differences were found in the percentage of patients with cystocele detected in sitting position versus supine position.

No significant differences exist between the supine and sitting positions in the measures of the ARJ in the defecation phase, suggesting that the maximal level of pelvic floor descent is more influenced by the muscles elasticity and by the pelvic floor muscle voluntary contractions than by the gravity force (Figures 2, 4(c), and 4(d)).

Although the percentage of patients with pathological dynamic descent did not significantly differ between the two procedures, a statistically significant difference was found comparing the grade of dynamic descent between supine and sitting positions. This is explained considering that in supine position pelvic organs are located more cranially in respect to the PCP than in sitting position whereas in defecation phase the values in evacuation do not significantly differ between the two positions of examination. So, the MR in supine position may overestimate the grade of the dynamic descent of the pelvic floor.

The existence of a significant linear correlation between the measures detected in supine versus sitting position for most of the considered measures will encourage further studies for the definition of new cut-off values to be adopted when examining the patients in supine position, since the cut-off values currently used are taken from studies on RX-Defecography, performed in sitting position [10].

It will be also of interest to investigate if the MR in sitting position allows one to improve the detection and the accuracy in diagnosing and grading pelvic pathologies (rectocele, pelvic floor hernias).

To our knowledge, this is the largest series of patients who underwent MR Defecography both in supine 1.5 T and in sitting 0.25 T magnets; a new prototype was used allowing one to obtain diagnostic quality of the images in all the examinations. The limit of the prototype is currently due to the width of the magnet, allowing one to image only patient with hip circumference less than 100 cm. This can be optimized in the future, once the accuracy of this new system is validated.

## 5. Conclusion

Our results show that MR Defecography in sitting position may represent a useful tool to correctly diagnose and grade the pelvic organ descent. This is of pivotal importance in the assessment of patients with pelvic floor disorders since it may help the surgeon in the definition of the appropriate surgical therapy.

## Figures and Tables

**Table 1:** Synthesis of the measures (in cm) of pelvic organs in respect to the PCP, in rest, squeeze, and defecation phases in both sitting and supine examinations in all patients. SD: standard deviation.

	Sitting position			Supine position		
	Rest	Squeeze	Defecation	Rest	Squeeze	Defecation
Bladder						
Mean (±SD)	9.29 (1.37)	1.72 (1.26)	−1.35 (1.78)	2.41 (0.9)	2.68 (0.88)	−0.41 (2.06)
Median (range)	1.40 (3.56–−2.72)	1.80 (4.70–−2.20)	−1.37 (1.90–−4.30)	2.34 (4.90–0.48)	2.51 (5.18–1.40)	−0.70 (3.23–−4.35)
Vagina						
Mean (±SD)	3.23 (0.79)	3.70 (0.76)	0.13 (2.61)	4.47 (0.87)	4.47 (0.87)	1.48 (2.45)
Median (range)	3.27 (4.48–0.74)	3.80 (5.25–1.94)	1.04 (3.20–−5.40)	4.49 (6.21–2.57)	4.49 (6.21−2.57)	2.14 (5.95–−4.32)
ARJ						
Mean (±SD)	−2.88 (1.05)	−1.51 (1.36)	−5.15 (1.99)	−1.45 (1.78)	−0.59 (1.78)	−4.72 (1.70)
Median (range)	−3.00 (−0.97–−5.30)	−1.66 (1.30–−4.86)	−5.08 (−1.00–−8.97)	−1.87 (4.19–−5.12)	−1.87 (4.19–−5.12)	−4.96 (−1.14–−8.56)

**Table 2:** Synthesis of the measures (in cm) of pelvic organs in respect to the PCP, in rest, squeeze, and defecation phases in both sitting and supine examinations in male and female patient subgroups. SD: standard deviation.

	Sitting position						Supine position					
	Rest		Squeeze		Defecation		Rest		Squeeze		Defecation	
	Male	Female	Male	Female	Male	Female	Male	Female	Male	Female	Male	Female
Bladder												
Mean (SD)	2.34 (0.73)	0.71 (1.28)	3.52 (1.06)	1.45 (±1.05)	0.36 (1.27)	−1.58 (±1.74)	4.11 (0.67)	2.15 (0.60)	4.37 (0.99)	2.43 (0.50)	2.20 (0.66)	−0.79 (1.91)
Median (range)	2.32 (3.15–1.59)	0.8 (2.15–−2.72)	3.59 (4.70–1.79)	1.63 (2.77–−2.20)	0.64 (1.70–−1.55)	−1.58 (1.90–−4.33)	4.33 (4.80–3.00)	2.28 (2.94–0.49)	4.82 (5.18–2.68)	2.45 (3.20–1.40)	2.04 (3.23–1.50)	−0.10 (2.15–−4.35)
ARJ												
Mean (SD)	−3.35 (1.05)	−2.81 (1.03)	−1.41 (0.23)	−1.52 (1.46)	−5.40 (2.01)	−5.11 (1.98)	−1.93 (0.61)	−1.38 (1.88)	0.25 (1.59)	−0.71 (1.45)	−3.97 (0.85)	−4.83 (1.77)
Median (range)	−3.04 (−2.34–−4.98)	−3.00 (−0.97–−5.30)	−1.47 (−1.04–−1.66)	−1.75 (1.30–−4.86)	−4.81 (−3.30–−8.70)	−5.20 (−1.00–−8.97)	−2.25 (−0.87–−2.35)	−1.80 (4.19–−5.12)	0.03 (2.26–−1.32)	−0.89 (1.78–−3.90)	−3.65 (−3.23–−5.34)	−5.00 (−1.14–−8.56)

**Table 3:** Pearson's test correlation coefficient and related *p* value (in parentheses).

	Sitting/supine		
	Rest	Squeeze	Defecation
All patients			
Bladder	**0.71** (8.07 · 10^−6^)	**0.854** (9.64 · 10^−10^)	**0.753** (1.04 · 10^−6^)
ARJ	0.228 (0.217)	**0.517 (0.0029)**	**0.696** (1.36 · 10^−5^)
Female subgroup			
Bladder	**0.678** (1.02 · 10^−4^)	**0.808** (3.35 · 10^−7^)	**0.806** (3.82 · 10^−7^)
Vagina	**0.611 **(6.77 · 10^−4^)	0.31 (0.176)	**0.796** (1.61 · 10^−5^)
ARJ	0.277 (0.162)	0.568 (0.002)	**0.805** (4.03 · 10^−7^)
